# Aberrant Hippocampal Network Connectivity Is Associated With Neurocognitive Dysfunction in Patients With Moderate and Severe Obstructive Sleep Apnea

**DOI:** 10.3389/fneur.2020.580408

**Published:** 2020-12-11

**Authors:** Li Zhou, Guiqian Liu, Hong Luo, Huabing Li, Yating Peng, Dandan Zong, Ruoyun Ouyang

**Affiliations:** ^1^Department of Pulmonary and Critical Care Medicine, The Second Xiangya Hospital, Central South University, Changsha, China; ^2^Research Unit of Respiratory Disease, Central South University, Changsha, China; ^3^Diagnosis and Treatment Center of Respiratory Disease, Central South University, Changsha, China; ^4^Hunan Province Prevention and Treatment Institute for Occupational Diseases, Changsha, China; ^5^Department of Radiology, The Second Xiangya Hospital, Central South University, Changsha, China

**Keywords:** obstructive sleep apnea, neurocognitive impairment, hippocampus, functional connectivity, cerebellum, default mode network

## Abstract

**Objectives:** This work aims to explore the changes of functional connectivity (FC) within the hippocampus network in patients with moderate and severe obstructive sleep apnea (OSA) and its correlation with neurocognitive dysfunction to explore the potential neurophysiological mechanism.

**Methods:** A total of 32 treatment-naïve patients with moderate or severe OSA and 26 healthy controls (HCs), matched in age, gender, and education, underwent the evaluations of Epworth Sleep Scale, neurocognitive function, full-night polysomnography, and resting-state functional magnetic resonance imaging. The FC map of the hippocampus to other brain areas was compared among 15 OSA patients and 15 HCs with little head motion. Finally, the correlation between hippocampus FC strength and respiratory sleep parameters and neurocognitive assessments was analyzed.

**Results:** Compared with HCs, the right hippocampus showed a significantly decreased FC with the bilateral insular lobe, right thalamus, and right anterior cingulate gyrus (ACG) and an increased FC with the right superior and middle temporal gyrus, left posterior cingulate gyrus, and left angular gyrus in the patients with OSA. The left hippocampus presented a significantly decreased FC with the left anterior cerebellum in patients with OSA. In addition, the aberrant right hippocampal FC with the right ACG was significantly correlated with disease severity and disrupted sleep architecture in the OSA group. Furthermore, after adjusting the related confounding factors, the FC strength between the right hippocampus, right insular lobe, and right thalamus was positively associated with the scores of Stroop Color–Word Test (SCWT) or Hopkins Verbal Learning Test—Revised (HVLT-R), while the FC between the right hippocampus and the right middle temporal gyrus was negatively correlated with the scores of HVLT-R. The right hippocampus FC with right superior temporal gyrus, left angular gyrus, and ACG were all negatively related to the scores of the symbol coding test (*r* = −0.642, *p* = 0.045; *r* = −0.638, *p* = 0.047; *r* = −0.753, *p* = 0.012), respectively. The FC between the left hippocampal and the left anterior cerebellar lobe showed a positive relationship with the scores of HVLT-R (*r* = 0.757, *p* = 0.011) and CPT-3D (*r* = −0.801, *p* = 0.005).

**Conclusion:** The hippocampus presented abnormal FC with the cerebral and cerebellar regions extensively in OSA, and the correlation between abnormal hippocampal network FC and neurocognitive dysfunction in OSA suggests a promising insight to explore the potential biomarker and pathophysiologic mechanism of neurocognitive dysfunction of OSA.

## Background

Obstructive sleep apnea (OSA) syndrome is one of the commonest types of sleep disorder, of which the prevalence of moderate-to-severe OSA in the population is high, with up to 49.7% in men and 23.4% in women (1). Repetitive partial or complete obstruction of the upper airway during sleep leads to two prominent pathophysiological characteristics of OSA: nocturnal intermittent hypoxia and sleep fragmentation/disruption (1). More importantly, chronic intermittent hypoxia and sleep disruption would cause a large amount of production in reactive oxygen species, inducing excessive activation of oxidative stress responses, including lipid peroxidation, protein oxidation, and DNA oxidation, and leading to dysfunction of the mitochondria, endoplasmic reticulum, and endothelial cell, plus massive inflammatory responses (2, 3). The brain, especially the cerebral cortex and hippocampus, is extremely vulnerable to hypoxia and oxidative stress, and the above-mentioned pathophysiological changes in the brain of OSA patients could induce the overproduction of neuroinflammatory cytokines and cellular dysfunction, resulting in chronic damage and even apoptosis of neuronal cells, and eventually lead to neurocognitive dysfunction (3). Neurocognitive impairment is a frequent clinical complaint from patients with OSA, which involves declined memory, attention/vigilance deficit, impaired executive function, etc. (4, 5).

In the recent decades, neuroimaging technologies, including functional magnetic resonance imaging (fMRI), voxel-based morphometry, magnetic resonance spectroscopy, diffusion tensor imaging, *etc*., were introduced in studies of OSA to investigate the changes of brain function and structures and explore the potential neuropathologic mechanism of neurocognitive impairment in OSA (6–9). The previous structure MRI studies found decreased white matter integrity and volume (10, 11), regional cortical thinning (12), altered gray matter volume and density (6, 13), reduced mean diffusivity (9), and abnormal cerebral metabolisms, such as regional reduced N-acetyl aspartate/choline ratios and choline/creatine ratios, lower γ-aminobutyric acid, and higher glutamate (7, 14), among OSA patients. Resting-state fMRI (rs-fMRI), an advanced fMRI technology to evaluate brain activity in the spontaneous state, has demonstrated abnormality in regional homogeneity (15), global and regional functional connectivity (FC) (16), and amplitude of low-frequency fluctuation (17) within individuals with OSA. rs-fMRI is a useful tool to detect the changes of brain functional activities in neurodegenerative diseases (18, 19), in which seed-based FC is a widely applied approach to evaluate the functional synchronicity between a specific region of interest and the rest of the brain voxels by calculating the correlation coefficients of blood oxygen level-dependent time series signals among these brain regions (20). Abnormal FC was found in multiple brain regions including the insula (16, 21), amygdala sub-regions (22), prefrontal cortex (23), and default-mode network (24–26) in OSA patients.

In recent years, more and more evidence demonstrated that OSA was an important risk factor for Alzheimer's disease (AD) (27, 28). AD is an irreversible neurodegenerative disorder characterized by a progressive decline in memory and other neurocognitive functions, including visual–spatial skills, attention, executive function, decision-making ability, language ability, personality and behavioral abnormality, *etc*. (29) Classical cerebrospinal fluid biomarkers of AD, such as increased tau proteins, reduced β-amyloid_42_, and elevated lactate levels, were demonstrated in the cerebrospinal fluid of individuals with OSA (30, 31). OSA also is involved in up-regulating the phosphorylation of tau proteins, promoting the production of β-amyloid_42_, and enhancing synaptic dysfunction, which indicate that similar pathophysiological alterations in the brain existed between AD and OSA (27). It is widely acknowledged that the hippocampus is one of the brain regions with the most prominent pathological lesions in patients with AD (29). The hippocampus is a pivotal and fundamental brain area responsible for neurogenesis and function of dentate gyrus and hippocampal circuitry, playing an important role in the process of learning and memory, including sensory memory, short-term memory, and long-term memory (32). Besides these, the hippocampus is vulnerable to hypoxia, oxidative stress, and inflammation, and these pathological responses are recognized as classical pathological manifestations of OSA (2). A brain MRI study conducted by Torelli and colleagues demonstrated that the total volume of the hippocampus was reduced in patients with moderate–severe OSA and was correlated with scores of the tests of verbal memory and executive function (33). However, little is known about hippocampal FC and its relationship with different fields of neurocognitive function in patients with OSA.

In this study, MATRICS Consensus Cognitive Battery and Stroop Color–Word Test (SCWT) were employed to evaluate the neurocognitive impairment in patients with moderate to severe OSA, including multiple cognitive fields in information processing speed, memory, attention/alertness, and executive function. Furthermore, the FC of the hippocampus network was measured by the method of seed point correlation analysis, and the correlation between FC strength and respiratory–sleep parameters and neurocognitive function was further analyzed.

## Materials and Methods

### Subjects

A total of 32 treatment-naïve, newly diagnosed patients with moderate to severe OSA [apnea hypopnea index (AHI) >15 events/h) from the respiratory and sleep center of The Second Xiangya Hospital, Central South University, and 26 age-, sex- and education years-matched healthy controls (HCs) were recruited in this study (Table 1). The diagnosis criteria of OSA referred to the guideline published by American Academy of Sleep Medicine in 2014 (34). The exclusion criteria for all subjects were as follows: [1] clinical history of heart disease, neurological disorder, psychiatric illness, other respiratory or sleep disorders, malignant tumors, drug and alcohol abuse, recent surgery, trauma, infection, or other system diseases, *etc*., [2] received the same or similar cognitive tests before, and [3] contraindicated for MRI. The inclusion criteria for HCs were as follows: [1] good sleepers, no snoring or apnea during sleep, [2] AHI <5 (confirmed by followed polysomnography), [3] body mass index (BMI) >24 kg/m^2^, and [4] without neurological diseases that can potentially influence the results of neurocognitive function and fMRI. This research protocol was approved by the Ethics Committee of The Second Xiangya Hospital, Central South University, and all subjects were informed of the study details and have provided their consent before participation.

**Table 1:** Comparison of characteristics in demography, clinical and sleep parameters, and neurocognitive tests between obstructive sleep apnea (OSA) group and HC group.

**Characteristics**	**OSA (*n* = 32)**	**HC (*n* = 26)**	***p***
Age (year)^a^	43.44 ± 11.14	42.77 ± 12.67	0.832
Men/women^c^	28/4	20/6	0.062
Education (year)^b^	12 (9–14)	11 (9–16)	0.762
BMI (kg/m^2^)^a^	28.87 ± 3.29	27.18 ± 1.68	0.015^*^
Smoking index^b^	65 (0–400)	0 (0–425)	0.402
Drinking index^b^	0 (0–825)	0 (0–250)	0.565
ESS scores^b^	13.78 ± 4.72	8.31 ± 4.23	<0.001^**^
Nocturnal SP (mmHg)^a^	135.94 ± 14.62	127.54 ± 14.57	0.034^*^
Nocturnal DP (mmHg)^b^	88.5 (81.8–93.8)	78 (72.0–83.5)	<0.001^**^
Heart rate (beats/min)^a^	94.3 ± 11.68	76.46 ± 7.61	<0.001^**^
AHI (events/h)^b^	60.40 ± 21.2	2.34 ± 1.45	<0.001^**^
ODI (events/h)^b^	61.75 (38.5–79.5)	1.65 (0.67–2.92)	<0.001^**^
LSaO_2_ (%)^b^	62.5 (54.5–75.0)	91 (88.3–93.0)	<0.001^**^
MSaO_2_ (%)^b^	94 (87.3–95.0)	96 (95–97)	<0.001^**^
Total sleep time (min)^b^	370.8 (286.0–455.8)	411.8 (311.5–435.5)	0.684
Sleep efficiency (%)^a^	66.1 ± 21.34	69.44 ± 12.17	0.457
N1 stage/TST (%)^b^	20.9 (12.9–30.0)	18.5 (10.5–27.1)	0.321
N2 stage/TST (%)^a^	54.87 ± 17.20	51.36 ± 11.87	0.396
Light sleep/TST (%)^b^	85.6 (68.3–93.3)	71.4 (62.4–81.6)	0.011^*^
N3 stage/TST (%)^b^	7.5 (0.5–24.7)	19.7 (8.4–25.8)	0.139
REM/TST (%)^b^	4.1 (1.7–7.5)	11.65 (6.68–15.15)	<0.001^**^
Mean head motion (cm)^b^	0.151 (0.08–0.24)	0.086 (0.08–0.113)	0.07^**^
TMT-A^a^	44.69 ± 15.39	33.62 ± 8.85	0.037^*^
HVLT-R^b^	20 (15–23)	24 (19–28)	0.015^*^
WMS-III: spatial span^b^	14.5 (12–17)	17 (14–19)	0.016^*^
NAB: mazes^a^	13.34 ± 5.88	17.08 ± 4.80	0.012^*^
Category fluency test^a^	19.7 ± 6.2	24.7 ± 6.6	0.004^**^
SCWT—word^b^	85 (63–93)	88 (80–100)	0.049^*^
SCWT—color–word^b^	28 (20–40)	41 (31–48)	0.003^**^

*HC, healthy control; AHI, apnea–hypopnea index; ODI, oxygen desaturation index; LSaO_2_, lowest oxygen saturation; MSaO_2_, mean oxygen saturation; BMI, body mass index; ESS, Epworth Sleepiness Scale; SP, systolic pressure; DP, diastolic pressure; TST, total sleep time; REM, rapid eye movement; TMT-A, Trail Making Test: part A; HVLT-R, Hopkins Verbal Learning Test—Revised; WMS-III, Wechsler Memory Scale-III; NAB, Neurological Assessment Battery: mazes; SCWT, Stroop Color–Word Test*.

*Sleep efficiency is calculated by dividing total sleep time by total bedtime*.

* *p < 0.05*;

** *p < 0.01*.

a *Student's t-test (data are shown as mean ± standard deviation)*.

b *Mann–Whitney U-test [data are shown as median (interquartile range)]*.

c *Chi-square test (data are presented as number of people)*.

### Assessment of Sleepiness, Neurocognitive Function, and Polysomnography

Daytime sleepiness was evaluated by the Epworth Sleep Scale (ESS), and neurocognitive function was assessed by the MATRICS Consensus Cognitive Battery and SCWT when the subjects got admitted. Seven domains of neurocognitive function were assessed in this study, including speed of information processing, attention/vigilance, working memory, executive function, short-term memory in verbal learning, ability to reasoning, and problem solving. The full-night polysomnography (Embla S4000; Medcare Technologies, Fuquay Varina, NC, USA) was performed on the first night. The detailed process of neurocognitive evaluation has been shown in our previously published paper (35). All the above-mentioned assessments were uniformly conducted by a professionally trained physician who was blinded to the clinical information of subjects. All the examinations were performed in the same order.

### rs-fMRI Data Acquisition

All the subjects underwent resting-state functional MRI on a Philips 3.0 Tesla MRI scanner. The participants were asked to wear headphones to decrease scanner noise and minimize head motion, breathe calmly, keep their eyes closed, and stay motionless without any specific thoughts. The functional image data were obtained by an echo-planar imaging (EPI) sequence, and the parameters were as follows: TR/TE = 2,000/30 ms, 33 slices, 64 × 64 matrix, 22 × 22 cm^2^ field of view, 90° flip angle, 0.4 cm thickness, and 0.6 mm gap. Each functional MRI scanning took 8 min and 8 s, and 240 volumes (total of 7,920 images) were acquired.

### Data Preprocessing

The Matlab-based Statistical Parametric Mapping (SPM8) software (Wellcome Department of Imaging Neuroscience, London, UK) was used to preprocess the rs-fMRI imaging data. To avoid the impact of interference signal brought by the initial adaptation of the magnetic environment, the first five brain volumes of the rs-fMRI were discarded, and the remaining 235 volumes were preprocessed as follows: [1] Firstly, correction of slice-timing and head motion was conducted. The subjects with head translation in any cardinal direction (x, y, z) of more than 1.5 mm were excluded. Finally, 15 OSA patients and 15 HCs were selected for further analysis of the difference of FC among the hippocampus network (Table 2); [2] Secondly, spatial normalization of images was performed in a standard EPI template of the Montreal Neurological Institute, and then the images were resampled to 3 × 3 × 3-mm^3^ voxels; [3] Thirdly, the standardized functional images were spatially smoothened with a 6-mm full-width at half-maximum Gaussian kernel and temporally band pass-filtered (0.01–0.08 Hz); and [4] Lastly, a linear regression was performed to decrease the influence of spurious covariates and signals from low-frequency synchronous oscillation.

**Table 2:** Correlation between sleep structure and neurocognitive tests among obstructive sleep apnea group [adjusting for age, education, body mass index (BMI), smoking years, and alcohol years].

***n* = 32**		**TST (min)**	**Sleep efficiency (%)**	**LS/TST (%)**	**REM/TST (%)**
TMT-A	*r* =	0.010	−0.017	−0.228	0.413
	*p* =	0.959	0.931	0.253	0.032^*^
HVLT-R	*r* =	−0.118	−0.122	−0.410	0.228
	*p* =	0.557	0.544	0.033^*^	0.252
Category fluency	*r* =	−0.489	−0.501	0.164	0.138
	*p* =	0.010^*^	0.008^**^	0.415	0.492

*The data were obtained by partial correlation analysis by adjusting the confounding factors of age, education, BMI, smoking index and drinking index; TST, total sleep time; REM, rapid eye movement; LS, light sleep; TMT-A, Trail Making Test: part A; HVLT-R, Hopkins Verbal Learning Test—Revised*.

* *p < 0.05*;

** *p < 0.01*.

### Analysis of FC Among Hippocampal Networks

The bilateral hippocampus was defined as the seed region of interest in accordance with the automated anatomical labeling template generated by Rest 1.8 software (http://www.restfmri.net/forum/REST_V1.8) (36). The left and the right hippocampus were selected as two seeds, and FC coefficients were calculated within the whole brain. The average blood oxygen level-dependent time series of the seed region of interest was extracted from each subject, and then the Pearson correlation coefficient was computed between the time series of the seed and the time series of each voxel in the whole brain. The FC value of each voxel in the whole brain represented the FC of this voxel with the seed region of interest, and the correlation coefficients were transformed into *Z* values by using Fisher *Z*-score to bring the results closer to a normal distribution. Two independent-sample Student's *t*-tests were performed to identify the brain regions that show a significantly different functional connection with the hippocampus between the OSA group and the HCs group, and the brain region with *p* < 0.001 and cluster volume > 10 voxels was defined as a meaningful brain region with significant FC difference in the hippocampus network after AlphaSim correction. Finally, the significantly different brain regions' images were presented by using the XjView software (http://people.hnl.bcm.tmc.edu/cuixu/xjView). In addition, the REST software was used to extract the FC strength between the peak point of the abnormal FC brain areas of each patient and the hippocampus, the correlation analysis between the aberrant FC coefficients and sleep–breathing parameters, and the scores of neurocognitive function tests within the OSA group.

### Statistical Analysis

Statistical analysis was conducted by using the SPSS 23.0 statistical software (IBM Corp., Armonk, NY, USA). Kolmogorov–Smirnov test was performed to evaluate the normality of data. Student's *t*-test was employed in the normally distributed data, and Mann–Whitney *U*-test was used to analyze the non-normally distributed data to compare the data difference among the OSA group and HCs group. Partial correlation analysis was used in all the correlation analyses by adjusting the potential confounding effects, including age, BMI, and education years, to analyze the relationship between abnormal hippocampal FC, sleep–breathing parameters, and neurocognitive impairment in the OSA group. *P* < 0.05 was considered significantly different. Double-sided test was employed among all data analysis.

## Results

### Demographic, Clinical, and Neurocognitive Function Data

As shown in Table 1, no significant differences were shown in gender, age, education years, smoking index, and drinking index between the OSA group (*n* = 32) and HCs group (*n* = 26). As expected, significant differences were found in body mass index, AHI, oxygen desaturation index (ODI), mean oxygen saturation (MSaO_2_), lowest oxygen saturation (LSaO_2_), and scores of ESS among these two groups. Significantly elevated nocturnal systolic blood pressure, diastolic blood pressure, and heart rate were also observed in patients with OSA. In addition, patients with OSA presented increased light sleep (LS) and decreased N3 sleep and rapid eye movement (REM) sleep. No significant differences were found in total sleep time, sleep efficiency, and N1 sleep and N2 sleep between the OSA group and the HCs group. Besides these, compared with HCs, significantly increased head motion was presented in the OSA group. Furthermore, the OSA patients showed poorer performance in the multiple neurocognitive tests including Trail Making Test A (TMT-A), Hopkins Verbal Learning Test—Revised (HVLT-R), Wechsler Memory Scale-III: spatial span (WMS-IIISS), mazes, category fluency, Continuous Performance Test (CPT), and SCWT (*p* < 0.05).

### Correlation Between Sleep Structure and Blood Pressure, Neurocognitive Tests in Patients With OSA

**Figure 1:**
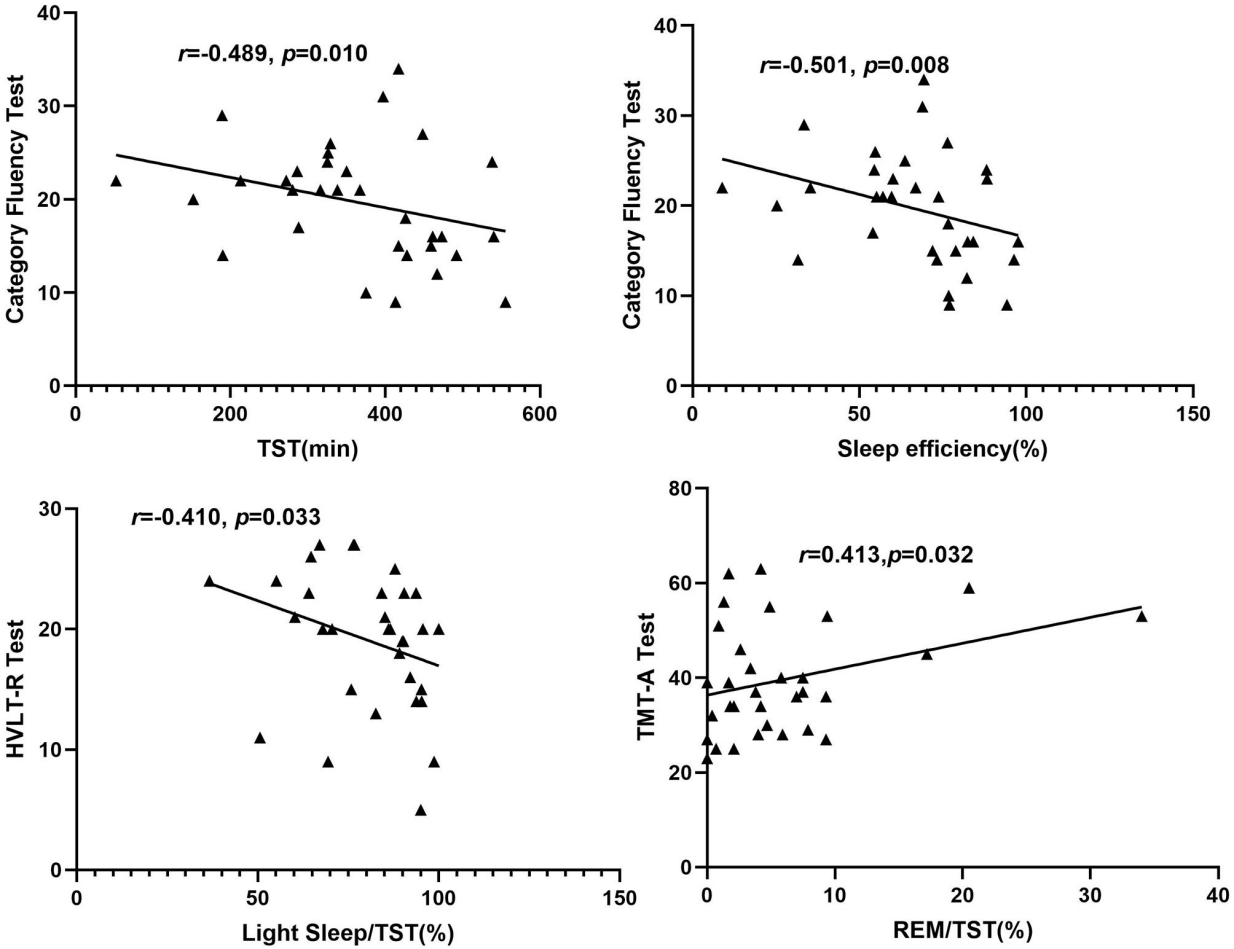
Correlation between sleep structure and neurocognitive tests among patients in the obstructive sleep apnea group. REM, rapid eye movement; TMT-A, Trail Making Test: part A; HVLT-R, Hopkins Verbal Learning Test—Revised.

Among the OSA group, after adjusting the confounding factors of age, education years, BMI, smoking index, and drinking index, the proportion of REM in TST (REM%TST) was positively associated with the scores of the TMT-A test (*r* = 0.413, *p* = 0.032), and the percentage of light sleep in TST (LS%TST) was negatively correlated with the scores of HVLT-R (*r* = −0.410, *p* = 0.033). The scores of the category fluency test were negatively correlated with TST (*r* = −0.489, *p* = 0.010) and sleep efficiency (*r* = −0.501, *p* = 0.008) (shown in Table 2, Figure 1).

### Alterations of FC Among Hippocampal Network in Patients With OSA

**Figure 2:**
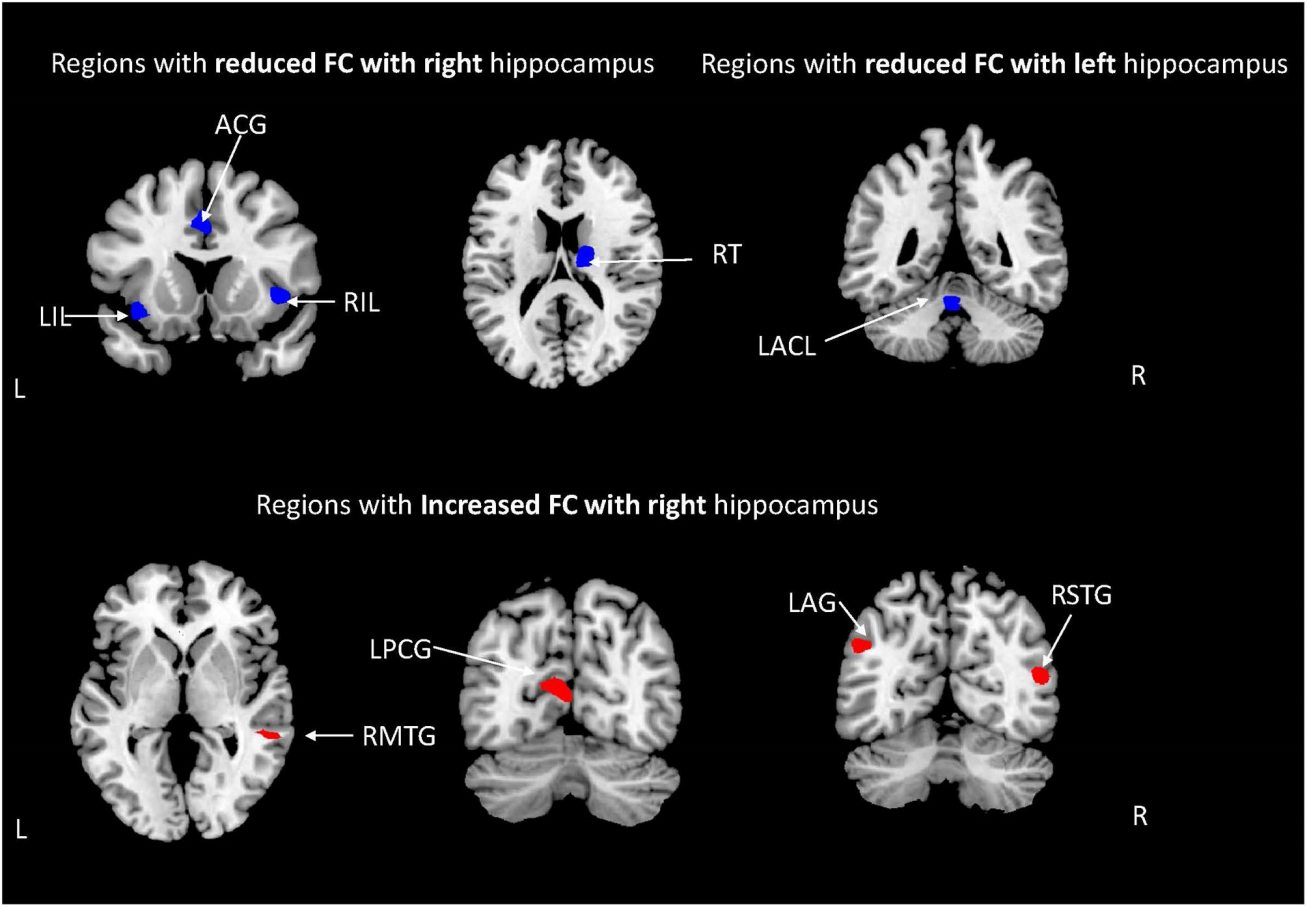
The brain regions with abnormal hippocampal functional connectivity (FC) in patients with moderate to severe obstructive sleep apnea [weakened FC (blue), enhanced FC (red); AlphaSim corrected; *P* < 0.001; cluster volume > 10 voxels].

The demographic characteristics of the subjects included in the analysis of hippocampal network FC are shown in Table 3. There was no significant difference in age, gender distribution, education years, BMI, smoking index, and drinking index between the OSA group and the HCs group. Compared with HCs, the right hippocampus presented a significantly reduced FC with the bilateral insular lobes, right thalamus, and anterior cingulate gyrus (ACG), while a significantly increased FC was observed with right middle temporal gyrus (RMTG), right superior temporal gyrus (RSTG), left posterior cingulate gyrus (LPCG), and left angular gyrus (LAG) in patients with OSA (shown in Table 4, Figure 2). In addition, the left hippocampus showed a decreased FC with the left anterior cerebellum lobes (LACL) in patients with OSA (Table 4, Figure 2).

**Table 3:** Basic clinical characteristics of the subjects included in the functional connectivity analysis.

	**Obstructive sleep**	**HC (*n* = 15)**	***P***
	**apnea (*n* = 15)**		
Age (year)^a^	42.87 ± 12.17	39.67 ± 12.52	0.484
Men/women^c^	13/2	12/3	0.775
Education (year)^b^	12 (9–16)	15 (9–16)	0.461
BMI (kg/m^2^)^a^	28.44 ± 3.38	27.16 ± 1.57	0.199
Smoking index^b^	0 (0–400)	0 (0–300)	0.838
Drinking index^b^	0 (0–500)	0 (0–0)	0.902
ESS scores^a^	12.4 ± 3.62	8.14 ± 4.03	0.006^**^
Nocturnal SP (mmHg)^a^	138.9 ± 16.31	124.8 ± 8.87	0.006^**^
Nocturnal DP (mmHg)^b^	92 (92–102)	75 (70–78)	<0.001^**^
Heart rate (beats/min)^a^	92.9 ± 12.55	77.1 ± 8.41	<0.001^**^
AHI (events/h)^a^	58.9 ± 22.74	2.17 ± 1.26	<0.001^**^
ODI (events/h)^a^	52.9 ± 25.21	1.61 ± 0.93	<0.001^**^
LSaO_2_ (%)^a^	67.2 ± 14.41	90.6 ± 3.06	<0.001^**^
MSaO_2_ (%)^b^	92 (94–96)	96 (95–97)	0.005^**^
Total sleep time (min)^b^	328.5 (190–447.5)	420 (333.5–432.5)	0.217
Sleep efficiency (%)^a^	58.1 ± 24.5	73.98 ± 8.45	0.029^*^
N1 stage/TST (%)^a^	32.6 ± 20.28	21.4 ± 11.81	0.075
N2 stage/TST (%)^a^	53.3 ± 19.24	50.3 ± 16.71	0.647
Light sleep/TST (%)^b^	58.1 ± 24.5	69.3 ± 10.56	0.120
N3 stage/TST (%)^b^	4.4 (0.3–11.8)	21.2 (10.8–24.5)	0.106
REM/TST (%)^b^	3.8 (1.3–7.5)	11.8 (5.4–18.7)	0.007^**^
Mean head motion (cm)^a^	0.1 ± 0.0315	0.078 ± 0.015	0.021^*^

*HC, healthy control; AHI, apnea–hypopnea index; ODI, oxygen desaturation index; LSaO_2_, lowest oxygen saturation; MSaO_2_, mean oxygen saturation; BMI, body mass index; ESS, Epworth Sleepiness Scale; SP, systolic pressure; DP, diastolic pressure; TST, total sleep time; REM, rapid eye movement*.

* p < 0.05 and

** *p < 0.01*.

a *Student's t-test (data are shown as mean ± standard deviation)*.

b *Mann–Whitney U-test [data are shown as median (interquartile range)]*.

c *Chi-square test (data are presented as number of people)*.

**Table 4:** Difference of FC between the hippocampus and other brain areas.

	**Brain area**	**Brodmann area**	**Voxel**	**Montreal Neurological Institute atlas coordinates**			***t*-value**
				**X**	**Y**	**Z**	
Reduced FC	LIL	47	22	−36	15	−9	−4.85
	RIL	47	25	42	15	0	−4.10
	RT	–	44	18	−12	18	−5.05
	ACG	32	87	0	21	39	−4.86
	LACL	–	16	−3	−48	−24	−4.28
Increased FC	RMTG	21	18	60	−39	0	3.91
	LPCG	31	45	−6	−69	12	4.11
	RSTG	39	54	51	−63	15	4.77
	LAG	39	18	−48	−63	33	4.44

*FC, functional connectivity; LIL, left insular lobe; RIL, right insula lobe; RT, right thalamus; ACG, anterior cingulate gyrus; LACL, left anterior cerebellar lobe; RMTG, right middle temporal gyrus; LPCG, left posterior cingulate gurus; RSTG, right superior temporal gyrus; LAG: left angular gyrus*.

### Correlation Between Changes of Hippocampal Network FC and Sleep Respiratory Parameters in Patients With OSA

**Figure 3:**
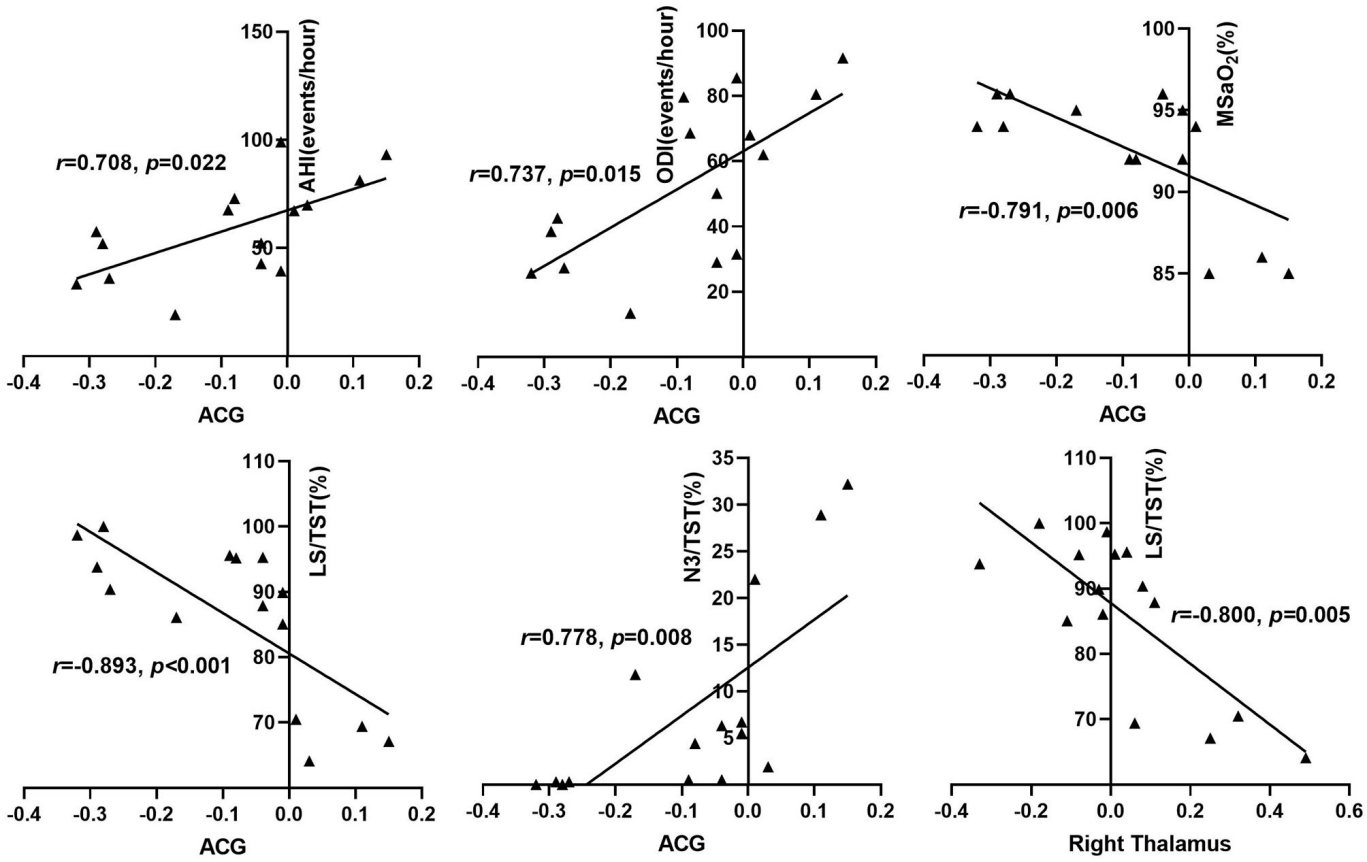
Correlation between reduced right hippocampus FC and sleep respiratory parameters among OSA patients. AHI, apnea-hypopnea index; ODI, oxygen desaturation index; MSaO2, mean oxygen saturation; TST, total sleep time; N3, stage 3 sleep; LS, light sleep; ACG, anterior cingulated gyrus.

**Figure 4:**
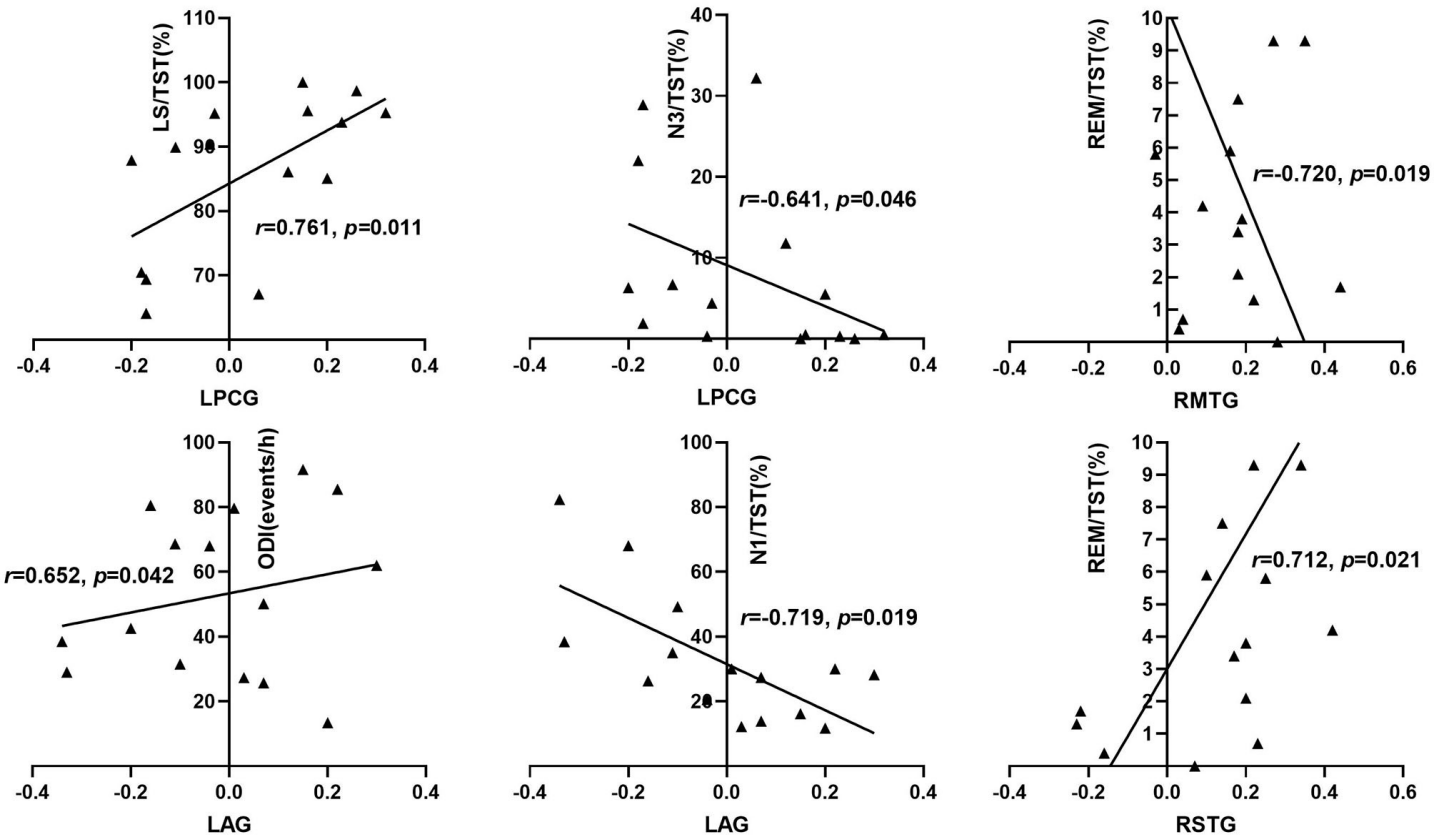
Correlation between increased right hippocampus FC and sleep respiratory parameters among OSA patients. ODI, oxygen desaturation index; ESS, Epworth sleep scale; TST, total sleep time; REM, rapid eye movement; N1, stage 1 sleep; N3, stage 3 sleep; LS, light sleep; LPCG, left posterior cingulate gyrus; RMTG, right middle temporal gyrus; RSTG, right superior temporal gyrus; LAG: left angular gyrus.

As shown in Table 5 and Figure 3, among the OSA group, after adjusting for the influence of age, education, BMI, smoking index, and drinking index, the FC strength between the right hippocampus and the right ACG was positively correlated with AHI (*r* = 0.708, *p* = 0.022), ODI (*r* = 0.737, *p* = 0.015), and N3 sleep %TST (*r* = 0.778, *p* = 0.008), respectively, but negatively correlated with mean SaO_2_ (*r* = −0.791, *p* = 0.006) and light sleep %TST (*r* = −0.893, *p* < 0.001). The FC strength between the right hippocampus and the right thalamus was negatively associated with light sleep %TST (*r* = −0.800, *p* = 0.005). The FC strength between the right hippocampus and the LAG showed a positive correlation with ODI (*r* = 0.652, *p* = 0.042) and a negative correlation with N1 sleep %TST (*r* = −0.719, *p* = 0.019) (Figure 4). The FC strength between the right hippocampus and RMTG was negatively associated with REM sleep %TST (*r* = −0.720, *p* = 0.019) and the scores of ESS (*r* = −0.650, *p* = 0.042). The FC strength between the right hippocampus and LPCG was positively associated with light sleep %TST (*r* = 0.761, *p* = 0.011) while negatively associated with N3 sleep %TST (*r* = −0.641, *p* = 0.046). The FC strength between the right hippocampus and RSTG was positively related to REM sleep %TST (*r* = 0.712, *p* = 0.021). In addition, the FC between the left hippocampus and LACL was positively related to REM sleep %TST (*r* = 0.817, *p* = 0.004).

**Table 5:** Correlation between hippocampus functional connectivity and sleep respiratory parameters among patients in the obstructive sleep apnea group.

***n* = 15**		**RMTG**	**LPCG**	**RT**	**RSTG**	**LAG**	**ACG**	**LACL**
AHI	*r* =	−0.080	−0.101	0.295	0.277	0.536	0.708	0.308
	*p* =	0.826	0.781	0.409	0.438	0.110	0.022^*^	0.387
ODI	*r* =	−0.023	−0.083	0.403	0.272	0.652	0.737	0.219
	*p* =	0.950	0.820	0.248	0.438	0.042^*^	0.015^*^	0.543
MSaO_2_	*r* =	0.239	0.416	−0.571	0.044	−0.467	−0.791	−0.265
	*p* =	0.506	0.232	0.085	0.904	0.174	0.006^*^	0.459
ESS	*r* =	−0.650	−0.043	0.323	−0.155	0.167	0.108	−0.155
	*p* =	0.042^*^	0.905	0.363	0.670	0.644	0.767	0.670
N1/TST (%)	*r* =	−0.033	0.089	−0.595	−0.181	−0.719	−0.615	−0.103
	*p* =	0.928	0.806	0.070	0.617	0.019^*^	0.058	0.776
LS/TST (%)	*r* =	0.254	0.761	−0.800	−0.122	−0.265	−0.893	−0.518
	*p* =	0.479	0.011^*^	0.005^**^	0.736	0.459	<0.001^**^	0.125
N3/TST (%)	*r* =	0.118	−0.641	0.585	−0.260	0.282	0.778	0.115
	*p* =	0.745	0.046^*^	0.076	0.468	0.430	0.008^**^	0.751
REM/TST (%)	*r* =	−0.720	−0.344	0.522	0.712	0.009	0.353	0.817
	*p* =	0.019	0.330^*^	0.122	0.021	0.980	0.317	0.004

*The data were analyzed by the method of partial correlation analysis with adjustment of the confounding factors of age, education, body mass index, smoking index, and drinking index*.

*AHI, apnea–hypopnea index; ODI, oxygen desaturation index; ESS, Epworth Sleep Scale; MSaO_2_, mean oxygen saturation; TST, total sleep time; REM, rapid eye movement; N1, stage 1 sleep; N3, stage 3 sleep; LS, light sleep; SP, systolic pressure; LPCG, left posterior cingulate gyrus; RMTG, right middle temporal gyrus; RT, right thalamus; RSTG, right superior temporal gyrus; ACG, anterior cingulated gyrus; LAG, left angular gyrus; LACL, left anterior cerebellum lobe*.

* p < 0.05 and

** *p < 0.01*.

### Correlation Between Changes of Hippocampal Network FC and Neurocognitive Dysfunction in Patients With OSA

**Figure 5:**
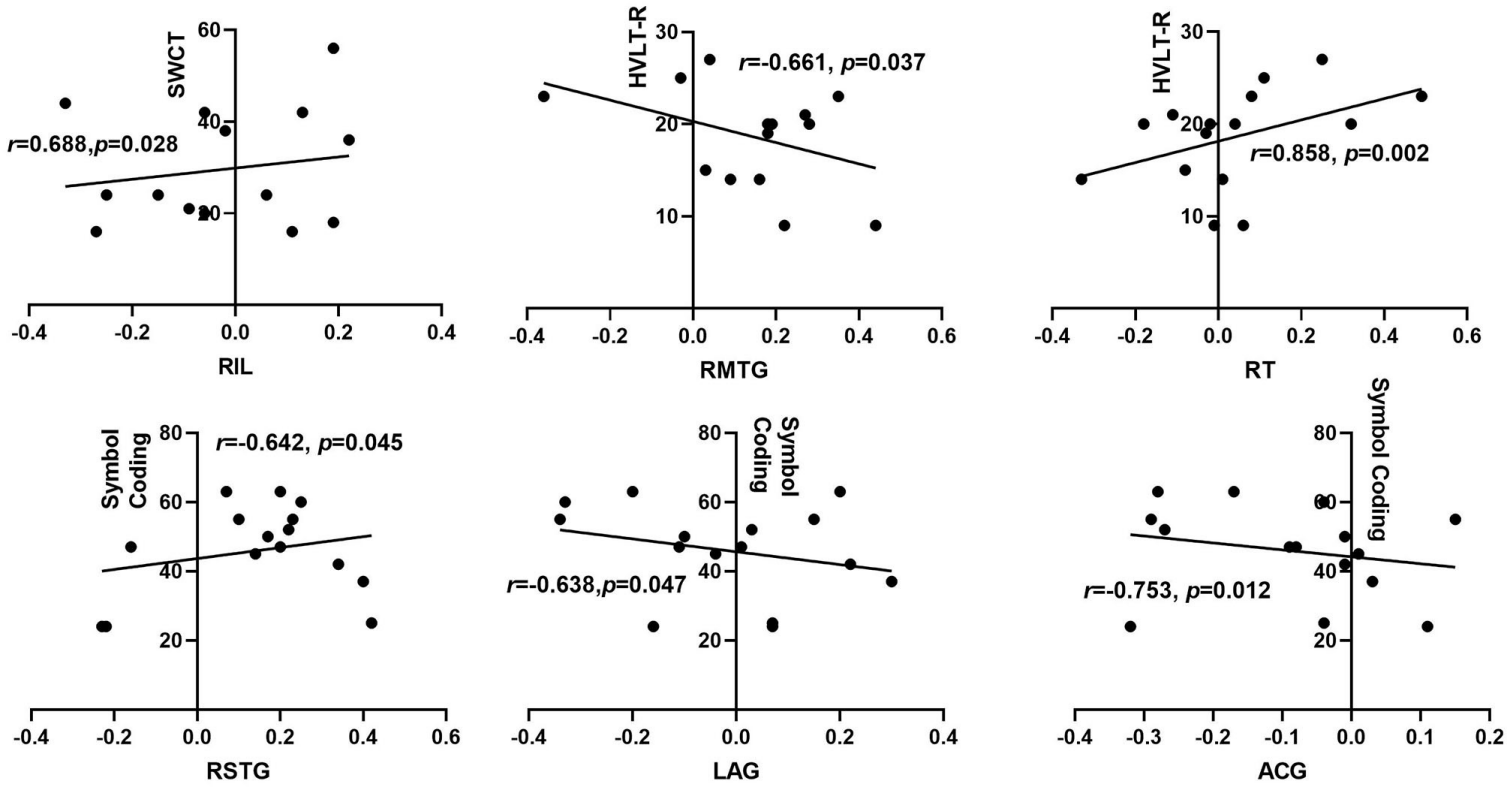
Correlation between right hippocampal FC and neurocognitive function among OSA patients. HVLT-R, Hopkins Verbal Learning Test-Revised; SCWT, Stroop color-word test; RIL, right insula lobe; RT, right thalamus; ACG, anterior cingulated gyrus; RT, right thalamus; RMTG, right middle temporal gyrus; RSTG, right superior temporal gyrus; LAG, left angular gyrus.

**Figure 6:**
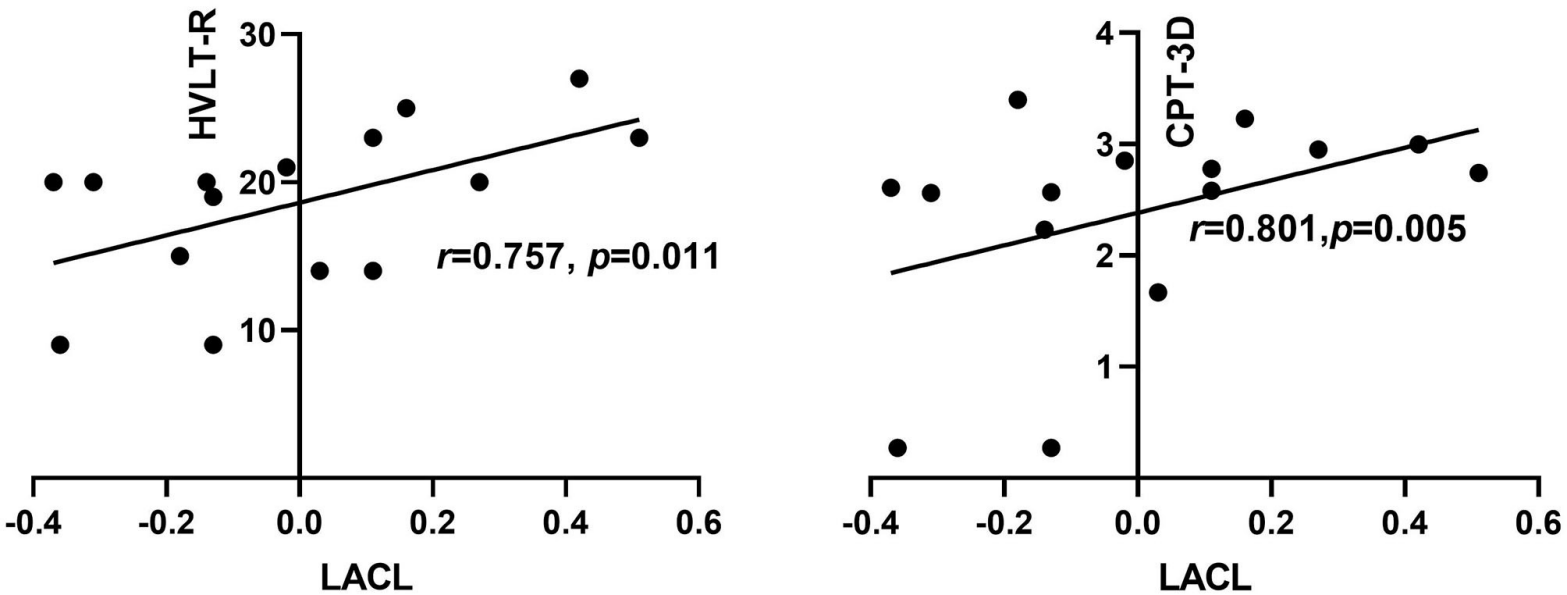
Correlation between left hippocampal FC and neurocognitive function among OSA patients. HVLT-R, Hopkins Verbal Learning Test- Revised; CPT, Continuous Performance Test; LACL, left anterior cerebellar lobe.

As shown in Table 6 and Figure 5, by adjusting the confounding variables of age, education, BMI, smoking index, and drinking index, the FC strength between the right hippocampus and the right insular lobe was positively associated with the scores of SCWT (*r* = 0.688, *p* = 0.028) within the OSA group. The FC strength between the right hippocampus and RMTG was negatively correlated with the scores of HVLT-R (*r* = −0.661, *p* = 0.037), while the FC strength between the right hippocampus and right thalamus was positively correlated with the scores of HVLT-R (*r* = 0.858, *p* = 0.002). The scores of the symbol coding test showed an inverse relationship with the FC strength between the right hippocampus and RMTG (*r* = −0.642, *p* = 0.045) and LAG (*r* = −0.638, *p* = 0.047), respectively. Moreover, for the left hippocampal FC, after adjusting the confounding factors of age, BMI, educational years, smoking index, and drinking index, the FC strength between the left hippocampus and LACL was positively related to the scores of HVLT-R (*r* = 0.757, *p* = 0.011) and CPT-3D test (*r* = 0.801, *p* = 0.005) (Figure 6).

**Table 6:** Correlation between bilateral hippocampal functional connectivity and neurocognitive function among obstructive sleep apnea patients.

***n* = 15**		**HVLT-R**	**Symbol coding**	**CPT-2D**	**CPT-3D**	**SWCT**
RIL	*r* =	0.214	−0.540	−0.261	0.381	0.688
	*p* =	0.552	0.107	0.466	0.277	0.028^*^
RMTG	*r* =	−0.661	0.037	−0.003	−0.619	0.120
	*p* =	0.037^*^	0.919	0.993	0.056	0.742
RT	*r =*	0.858	−0.372	−0.408	0.269	−0.255
	*p =*	0.002^**^	0.290	0.242	0.452	0.476
RSTG	*r* =	0.573	−0.642	−0.351	0.358	0.204
	*p* =	0.084	0.045^*^	0.319	0.309	0.571
LAG	*r* =	0.509	−0.638	−0.042	0.104	0.030
	*p* =	0.133	0.047^*^	0.909	0.775	0.935
ACG	*r* =	0.510	−0.753	−0.252	0.053	0.059
	*p* =	0.132	0.012^*^	0.482	0.884	0.871
LACL	*r* =	0.757	−0.206	−0.351	0.801	0.068
	*p* =	0.011^**^	0.567	0.321	0.005^**^	0.852

*The data were analyzed by the method of partial correlation analysis with adjustment of the confounding factors of age, education, body mass index, smoking index, and drinking index*.

*HVLT-R, Hopkins Verbal Learning Test—Revised; CPT, Continuous Performance Test; SCWT, Stroop Color–Word Test; RIL, right insula lobe; RT, right thalamus; ACG, anterior cingulated gyrus; LACL, left anterior cerebellar lobe; RT, right thalamus; RMTG, right middle temporal gyrus; RSTG, right superior temporal gyrus; LAG, left angular gyrus*.

* p < 0.05 and

** *p < 0.01*.

## Discussion

### FC Abnormality in the Hippocampal Network in OSA

The hippocampus, an important component of the limbic system, is located between the thalamus and the medial temporal lobe and plays a key part in learning, episode memory, and transformation of long-term memory (37); it is vulnerable to hypoxia and oxidative stress, which are important pathophysiological characteristics of OSA and thus may cause the impairment of synaptic plasticity and reduction of neurogenesis in the hippocampus (38). OSA is a significant risk factor and closely correlated with the onset of AD. The hippocampus is the commonest brain region suffering from the accumulation of β-amyloid_42_ and neurofibrillary tangles (27). The previous studies have demonstrated that OSA patients showed reduced gray matter volume in the hippocampus which significantly increased after treatment (8, 39). In the current study, we found that, compared with HCs, the right hippocampus had an extensively abnormal FC with the cerebral cortex, sub-cortex, and cerebellum, including the bilateral insular, right thalamus, cingulate gyrus, right temporal gyrus, and angular gyrus, in patients with moderate-to-severe OSA, and a significantly reduced FC was shown between the left hippocampus and the left anterior cerebellar lobe in the subjects with OSA. In a previous study, Song and his colleagues also found that the right hippocampus showed reduced FC with the bilateral thalamus and para-hippocampal gyrus in patients with OSA and enhanced FC with precuneus and posterior cingulate gyrus (40) which, to some degree, were consistent with the findings in our study. The hippocampus, medial temporal lobe, cingulate gyrus, and angular gyrus are crucial components of default mode networks (DMN), which play an important role in regulating emotion, consciousness, memory, and introspection (24). The DMN consists of two spatially independent sub-networks: the anterior DMN and the posterior DMN. The anterior DMN is comprised of the medial prefrontal cortex, superior frontal gyrus, and anterior cingulate gyrus, and the posterior DMN includes the precuneus and posterior cingulate gyrus. Research indicated that the anterior DMN was responsible for emotional management and self-reference, while the posterior DMN mainly contributed to cognitive processing and memory retrieval (41). In this study, the right hippocampus showed weakened FC with the anterior DMN and increased FC with the posterior DMN in patients with OSA, which suggests a possible role of the connectivity difference between the hippocampus and the anterior and posterior DMN in the formation of neurocognitive deficits of OSA and is likely to be a potential neurocognitive mechanism in the development of cognitive dysfunction in OSA.

However, it should be noted that there was a significant difference both in the nocturnal systolic pressure and the diastolic pressure between OSA group and HCs group in the current study, which are common manifestations in OSA due to the interaction between nocturnal intermittent hypoxemia, hypercapnia, sleep fragmentation, neurohormonal dysregulation, and sympathetic activation (42). Recent studies demonstrated the impact of hypertension on cognitive impairment and FC alterations (43, 44). The study by Li and his colleagues indicated that impaired attention and executive function existed in patients with hypertension, and furthermore, altered FC in the frontoparietal networks mediated the effects of changes of white matter on the decreased executive function in patients with hypertension (43). Similarly, Carnevale et al. also found that the altered FC network was associated with cognitive impairment and brain microstructural injury in patients with hypertension (44). The pathogenesis of OSA and hypertension is interactional and bidirectional, in which OSA is a crucial risk factor of hypertension and hypertension is a very common complication in OSA (45). The prevalence of OSA in hypertension ranges from 30 to 83% (46), and the prevalence of hypertension in OSA is also high (30–70%) (47). Furthermore, cardiovascular morbidity in OSA could further aggravate neurocognitive impairment (48). Hypertension and OSA could cause abnormal metabolism and perfusion of the hippocampus and cortex and further trigger brain injuries and related pathological changes of Alzheimer's disease (27). Therefore, it is difficult to isolate the effects of hypertension on the changes of FC and cognitive impairment in OSA patients. The consequences of abnormal brain FC and abnormal neurocognitive functions are probably caused by the combined effects of OSA and hypertension.

### FC Abnormality and Nocturnal Intermittent Hypoxia, Sleep Disturbance in OSA

In this study, after adjusting the confounding factors of age, education, BMI, smoking, and alcohol drinking index, we found that the FC between the right hippocampus and the anterior cingulate gyrus was positively correlated with AHI and ODI and negatively correlated with mean SaO_2_ among the OSA group, which indicated that chronic intermittent hypoxemia may play a role in the abnormality of FC within these two brain regions. The disrupted FC between bilateral hippocampus and anterior cingulate gyrus was also observed in patients with AD (49). Zhang et al. found that the FC within the anterior DMN network was related to AHI and considered that anterior DMN dysfunction in OSA patients may be associated with intermittent hypoxia and enhanced FC in the posterior DMN may be responsible for functional compensation in the brain (23). The hippocampus is susceptible to attacks of hypoxia and oxidative stress that are critical pathological features of OSA. The coordinated interaction between the hippocampus and the anterior cingulate gyrus is mainly involved in information processing, memory consolidation, and formation of recent and long-term memory (50, 51), which suggests that a possible correlation may exist between the abnormal hippocampus FC with the anterior cingulate and neurocognitive dysfunction in OSA. In addition, we found that the changes of hippocampus FC in other brain regions were also closely associated with the disturbance of sleep architecture of patients with OSA. Disturbed sleep structure is a prominent feature of patients with OSA, mainly manifested as typically increased light sleep (including N1 and N2 stage sleep) and reduced/absent deep sleep (including N3 and REM sleep) (52). The features of sleep structure of OSA patients in this study were consistent with the previous findings, which presented with increased light sleep stage and decreased N3 and REM sleep (Table 1). The disruption of sleep rhythm could result in disrupted synaptic homeostasis, metabolic dysfunction, excessive oxidative stress, neuro-inflammation, and decreased clearance of brain's metabolites and thus accelerates the pathogenesis of neurodegenerative disorders (53).

In the current study, the right hippocampus FC with the right thalamus or anterior cingulate gyrus both showed a negative correlation with light sleep %TST, while the right hippocampus FC with the left posterior cingulate gyrus showed a positive correlation with light sleep %TST. As we have described before, the right hippocampus revealed a reduced FC with both the right thalamus and the anterior cingulate gyrus but an enhanced FC with the posterior cingulate gyrus. It means that the elevated proportion of light sleep %TST was related to the reduced right hippocampus FC with the right thalamus and the anterior cingulate, a part of the anterior DMN, and the functional compensation between the right hippocampus and the posterior cingulate, a part of the posterior DMN.

It is widely acknowledged that N3 sleep and REM sleep have a fundamental part in the physiological functions of the hippocampus such as memory consolidation, memory re-activation, synaptic homeostasis, *etc*. (54) In this study, the proportion of N3 sleep %TST was positively correlated with the FC between the right hippocampus and the anterior cingulate gyrus and negatively correlated with the FC between the right hippocampus and the left posterior cingulate gyrus in the OSA group. These findings were coincident with the results of the FC changes between the right hippocampus and the cingulate gyrus, indicating that reduced N3 sleep was involved in DMN–hippocampal functional abnormality by disrupting the right hippocampus functional connection with the anterior DMN and enhancing the functional connection within the posterior DMN. N3 sleep, also known as slow wave sleep, plays a key role in declarative memory consolidation, re-activation of memory traces, clearing metabolites of the brain, orchestrating synaptic downscaling, and regulation of attention (55). The cingulate–hippocampal functional connection is involved in memory, learning, information processing, and emotion activity (56). We consider that disrupted N3 sleep may have an impact on the alteration of the right hippocampus FC with cingulate gyrus, which is likely to be a part of the pathophysiological mechanism for the pathogenesis of neurocognitive impairment in OSA.

With regards to the role of REM sleep in the changes of hippocampus FC, we found that the FC between the right hippocampus and the right middle temporal gyrus was negatively related to the percentage of REM sleep %TST in patients with OSA, while the FC between the right hippocampal and the right superior temporal gyrus was positively related to the percentage of REM sleep %TST. It has been mentioned in the previous paragraph that increased functional connection presented between the right hippocampus and the right middle and superior temporal gyrus in the OSA group. This means that, when the proportion of REM sleep in TST was high, compensation of FC between the right hippocampus and the right superior temporal gyrus would be strong, and when the percentage of REM sleep in TST was less, the compensational FC between the right hippocampus and the right middle temporal gyrus would become strong. This indicates that the proportion of REM sleep may have no effect on the overall functional connection strength between the right hippocampus and the right temporal lobe. REM sleep is mainly responsible for procedural memories consolidation and emotional memories consolidation (57), while the hippocampal–temporal functional connection is mainly involved in declarative memory and recognition memory such as recollection and familarity (58). The above-mentioned differences between REM sleep and hippocampal–temporal functional interaction in roles in memory reasonably explain the findings involving the relationship between REM sleep and increased FC between the right hippocampus and the right temporal gyrus in patients with OSA. The mechanism resulting in the enhanced hippocampal–temporal gyrus and its value in neurocognitive function in OSA remain to be further explored.

In summary, the above findings manifested that intermittent hypoxemia and sleep fragmentation may be involved in the abnormality of hippocampal FC with other regions. We speculated that FC damage between the right hippocampus and anterior cingulate gyrus may result from joint effects of nocturnal intermittent hypoxia and disturbance of sleep architecture. Meanwhile, reduced left hippocampus FC and the compensation of FC between the right hippocampus and the temporal gyrus were more likely to result from disruption of REM sleep, which further provides a new insight to explore the potential mechanism of neurocognitive impairment in OSA.

### Hippocampla FC Abnormality and Neurocognitive Dysfunction in OSA

In this study, we found that sleep parameters including total sleep time, sleep efficiency, light sleep, and REM sleep were closely related to the performance of information processing speed and memory in the OSA group (Table 2). The scores of category fluency were negatively correlated with total sleep time and sleep efficiency, indicating that sleep promoted cognitive dysfunction in patients with OSA. We consider that a large amount of sleep fragmentation and low-quality sleep cause the above-mentioned phenomenon in OSA. The proportion of light sleep in total sleep time was negatively correlated with the scores of memory tests, and the proportion of REM sleep in TST was positively associated with the scores of information processing speed test, indicating that disrupted sleep structure was involved in neurocognitive dysfunction in patients with OSA. In the previous section, it has been demonstrated that disrupted sleep architecture was involved in the abnormality of hippocampus FC with other brain regions in OSA. Combined with the above-mentioned findings, we speculated that abnormal hippocampus FC was involved in the pathogenesis and development of neurocognitive impairment in patients with OSA.

As expected, we found that the FC strength between the right hippocampus and the right insular lobe and thalamus were positively correlated with the performance of memory and executive function in the OSA group, in which the FC between the right hippocampus and the right insular lobe and thalamus were reduced compared with that in the HCs group. It indicates that the functional deficiency in these brain regions probably participates in the development of the impairment of memory and executive function in OSA. Meanwhile, the FC strength between the right hippocampus and the right superior temporal gyrus and left angular gyrus was negatively correlated with the performance of information processing speed, and the FC strength between the right hippocampus and the right middle temporal gyrus was also negatively correlated with the performance of memory in patients with OSA, in which the FC within these brain regions was enhanced compared with that of HCs. This suggests that an increased FC between the right hippocampus and other brain regions has not shown beneficial effects on alleviating neurocognitive impairment. The possible explanation for this finding is that the functional compensation between the right hippocampus and the left angular gyrus and right temporal gurus is incomplete to be able to prevent neurocognitive decline in patients with OSA. On the other hand, the FC abnormality within these brain areas probably promoted the neurocognitive dysfunction of OSA.

Another novel finding in our study is that we found that the left hippocampal FC with the left anterior cerebellum was significantly declined in patients with OSA compared with those in HCs. The FC between the left hippocampal FC and the left anterior cerebellum lobe was positively correlated with the percentage of REM sleep %TST, demonstrating that REM sleep might play a role in regulating the hippocampus functional interaction with the cerebellum. Besides these, the FC between the left hippocampus and the left anterior cerebellum showed a positive relationship with the performance of memory and attention test. To our current knowledge, this is the first time to discover that abnormal FC between the hippocampus and the cerebellum might be involved in neurocognitive impairment in patients with OSA. The hippocampal–cerebellar interaction has been demonstrated to play an important role in procedural memory, episodic memory, emotional memory, and information processing (spatial and temporal processing) (59). Previous studies have demonstrated that, compared with HCs, the gray matter volume and the density of cerebellum in patients with OSA were significantly reduced, and it was considered that intermittent hypoxia and sleep fragmentation may result in cerebellar structural changes (13, 60). We speculated that the disruption of REM sleep may, to some extent, cause the functional disconnection between the hippocampus and the cerebellum and contribute to declined memory and attention in the long term in OSA. The cerebellar–hippocampal functional disconnection may be a promising and novel mechanism of neurocognitive impairment of OSA.

All of above-mentioned findings suggest that FC abnormalities between the hippocampus and other brain regions may contribute to declined attention, executive function, and memory in patients with OSA. The hippocampus is extremely sensitive to hypoxia and oxidative stress. The typical pathophysiological alterations in OSA, including intermittent hypoxia/re-oxygenation, hypercapnia, changes of cerebral blood perfusion, and sleep fragmentation, could cause an accumulation of harmful metabolites, oxidative stress products, and neuro-inflammation cytokines (61), further resulting in functional disruption, pathological activities, and even structural changes in the hippocampus, ultimately leading to neurocognitive dysfunction.

However, there are some limitations of this study: Firstly, the sample size of this study is small, which may restrain the representativeness of the above-mentioned results. In order to minimize the effect of head movement on FC, we used a stricter inclusion criterion for head movement to obtain more reliable results, which led to the fact that a small number of OSA patients were included in this study. Secondly, this study was a clinical observational study with correlation analysis which cannot conclude an exact mechanism for neurocognitive impairment. Thirdly, patients with mild OSA were excluded from this study, which might have prevented us from generalizing the results to the whole population of OSA. Fourthly, moderate–severe OSA patients with a complication of hypertension or nocturnal hypertension were not excluded in this study. Hypertension is extremely common in OSA, with a high prevalence of up to 70%. Finally, due to a much lower prevalence in women, a much lower population of female patients was involved in the current study, which might weaken the generalizability of the findings to the whole population.

## Conclusion

Abnormal FC and adaptive compensation were demonstrated between the hippocampus and the cerebrum and cerebellum in patients with moderate to severe OSA. The right hippocampus mainly showed an altered FC with the cerebral cortex, sub-cortex, or DMN, while the left hippocampus mainly presented a changed FC with the left anterior cerebellar lobe. Moreover, this extensive abnormality of the right hippocampal FC may result from the joint effect of intermittent nocturnal hypoxia and sleep fragmentation, and the reduced FC between the left hippocampus and the anterior cerebellar lobe was more likely caused by a disturbed sleep architecture. More importantly, the abnormality of hippocampal network FC was closely related to neurocognitive impairment of OSA, including the cognitive fields of memory, attention, and executive function, which indicated a promising insight to explore the potential imaging biomarker and pathophysiologic mechanism of neurocognitive dysfunction of OSA.

## Data Availability Statement

The original contributions generated for the study are included in the article, further inquiries can be directed to the corresponding author.

## Ethics Statement

The studies involving human participants were reviewed and approved by Ethics Committee of The Second Xiangya Hospital, Central South University. The patients/participants provided their written informed consent to participate in this study.

## Author Contributions

LZ designed, conducted the study, and was a major contributor in writing the manuscript. GL contributed to data analysis. RO supervised, designed the study, and revised the manuscript. The rest of the co-authors contributed to data collection. All authors contributed to the article and approved the submitted version.

## Conflict of Interest

The authors declare that the research was conducted in the absence of any commercial or financial relationships that could be construed as a potential conflict of interest.
